# Shaping lace: Machine embroidered metamaterials

**DOI:** 10.1145/3639473.3665792

**Published:** 2024

**Authors:** Kate Glazko, Alexandra Portnova-Fahreeva, Arun Mankoff-Dey, Afroditi Psarra, Jennifer Mankoff

**Affiliations:** University of Washington, USA; CREATE, University of Washington, USA; Nova H.S., USA; Digital Arts & Experimental Media (DXARTS), University of Washington, USA; CREATE, University of Washington, USA

**Keywords:** Embroidery, metamaterials, accessibility

## Abstract

The ability to easily create embroidered lace textile objects that can be manipulated in structured ways, i.e., metamaterials, could enable a variety of applications from interactive tactile graphics to physical therapy devices. However, while machine embroidery has been used to create sensors and digitally enhanced fabrics, its use for creating metamaterials is an understudied area. This article reviews recent advances in metamaterial textiles and conducts a design space exploration of metamaterial freestanding lace embroidery. We demonstrate that freestanding lace embroidery can be used to create out-of-plane kirigami and auxetic effects. We provide examples of applications of these effects to create a variety of prototypes and demonstrations.

## 1 INTRODUCTION

**Figure 1:**
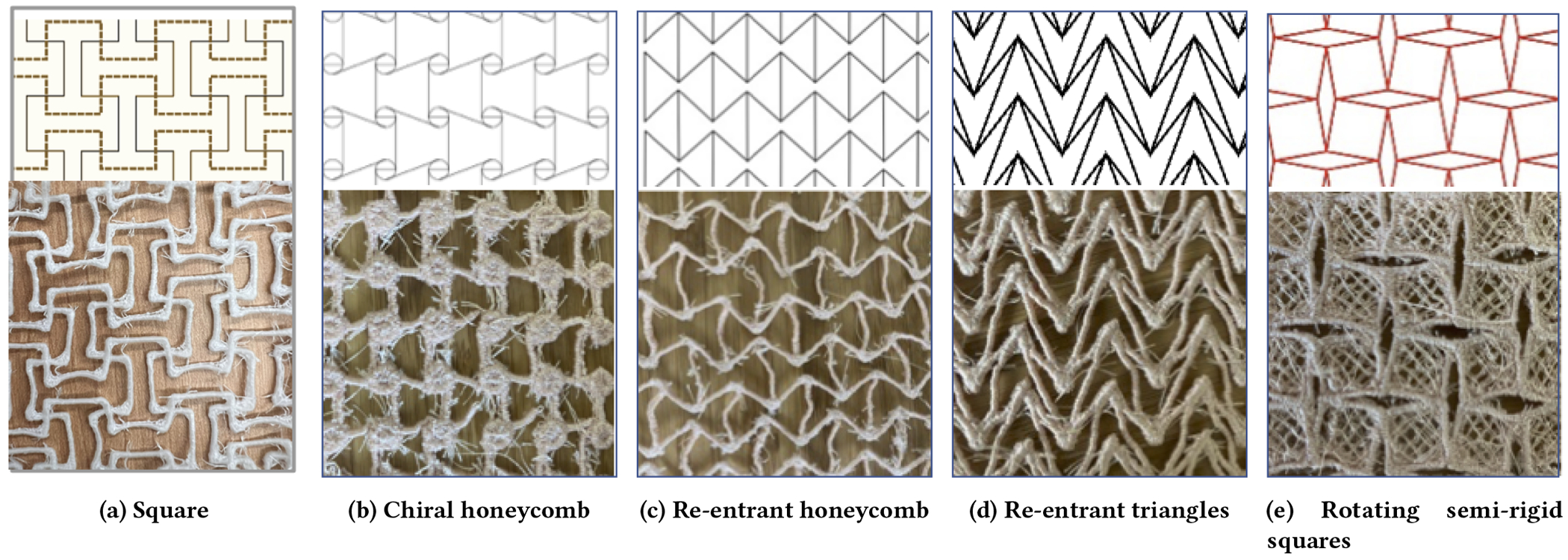
The different categories of auxetic structures we experimented with including the SVG model (top) and prints of the same structures (bottom). The Chiral honeycomb did not print successfully (as shown in Figure 2c)

Interest in metamaterials [Engheta and Ziolkowski 2006] has grown rapidly over the past two decades, reaching over 2,500 publications on the topic in recent years [Askari et al. 2020]. Metamaterials can be made out of almost anything, but they share a common property: their structure conveys mechanical or other properties rarely observed in naturally occuring materials. Metamaterials may add flexibility to stiff objects in various predictable ways [Bertoldi et al. 2017], including through the use of auxetic structures, which grow in both dimensions when stretched, as in Figure 1(a–d); and kirigami inspired folding structures [Callens and Zadpoor 2018], a Japanese art form that combines cutting and folding to create 3D shapes from planar prints, which are printed flat and twisted and rise up when untwisted.

Metamaterials support a variety of capabilities from sensing [Gong et al. 2021] to digitizing [Ion et al. 2017] to mechanics [Ion et al. 2016]. Among this body of work, metamaterial textiles have received considerable attention [Wang and Hu 2014] due to their numerous potential applications. Smart bandages can release medication based on swelling [Wang and Hu 2014]. Auxetic textiles can be used to line a prosthetic socket [Kowalczyk and Jopek 2020], to improve sports apparel [Moroney et al. 2018], and to create protective garments [Sun et al. 2021]. They have also been used to improve air flow, comfort, and thermal protection over conventional options [Ali et al. 2018; Lueling and Beuscher 2019].

Textile metamaterials have been manufactured using a variety of additive approaches [Askari et al. 2020] including weaving (e.g., [Cao et al. 2019; Kamrul et al. 2020; Zulifqar et al. 2018]) and machine knitting (e.g., [Hu et al. 2011; Knittel et al. 2015; Liu et al. 2010; Ma et al. 2017, 2016; Ugbolue et al. 2011]). However, access to technologies such as machine knitting remains limited and difficult to scale, and weaving is difficult to automate. This paper adds to that body of work by demonstrating the use of commercially-available computerized embroidery/sewing machines to create freestanding lace metamaterials. Embroidery machines are an inexpensive home manufacturing option available on mass-market commercial retail sites [Amazon 2023] for a few thousand dollars and are already a ubiquitous presence in many households and in community spaces (*e.g.* libraries [LAPL 2023a,b; of South SF 2023] and maker spaces). Additionally, they are more affordable than other types of textile manufacturing equipment, such as a computerized knitting machines, which can start in the range of thousands to hundreds of thousands of dollars.

In addition to being affordable and accessible, machine embroidery has several key differentiators as a means of production for textile metamaterials. Freestanding lace can be integrated with a wide range of fabrics and materials; allowing for easy embedding into clothing, sports gear, and more; allowing only specific parts of a design to have metamaterial properties. This selective embedding allows for the creation of prototypes that have increased aesthetics or subtlety compared to alternatives. Additionally, the ability to create freestanding lace metamaterial designs made of conductive threads allows for increased interactivity– some examples being the ability to create functioning antennas capable of transmitting information on touch [Kaputa and Psarra 2023a] and the ability to integrate capacitive sensing into lace [Jo and Kao 2021].

### Overview of Paper.

We conducted a design space exploration of auxetics and kirigami patterns to understand the capabilities of freestanding lace in both of these areas. We ran an empirical experiment measuring properties including strength and plastic deformation in various auxetic free-standing lace patterns to assess how the designs perform in a variety of conditions and to gain better insight into which different auxetic patterns are best suited for various applications. Based on the outcomes of the design space exploration, we created a series of design prototypes of functional objects demonstrating the uses of embroidered metamaterials. These prototypes showcase the unique attributes of embroidered metamaterials, such as theirstrength, ability to be integrated into other sewn contexts, and utility for creating digital circuits. The prototypes highlight potential for increased interactivity and customization, which we explore in this paper as the earliest stages of a research through design process [Stappers and Giaccardi 2017]. The chosen prototypes were selected based on potential promising applications from existing literature, as well as fulfilling practical needs in our own lives.

Our contributions are as follows:

- We replicate a range of auxetic metamaterials and prove that they are auxetic. We use these materials to create functional design prototypes of objects including a moldable fabric; accessible exercise band; fidgets; a cup holder; and a glucose monitor cover.
- We create kirigami objects that exhibit out-of-plane behavior using a combination of cuts, folds, and twists [Callens and Zadpoor 2018]. We use conductive thread and leverage out-of-plane twists and cuts to create a digital button; we use similar techniques to create a textile antenna.

The next section surveys existing literature in interactive textiles and introduces key concepts for metamaterials. Following that, we discuss the design space for auxetic metamaterials that we explored and our empirical experiments demonstrating which embroidered patterns exhibit auxetic properties. We then present functional design prototypes using both auxetic and out-of-plane machine embroidered metamaterials and demonstrate the applied value of these materials.

## 2 BACKGROUND

Metamaterials have the potential to support a variety of capabilities that enable novel forms of user interactions with materials [Gong et al. 2021], or simplify machines traditionally relying on complex electronics or circuitry [Ion et al. 2016, 2017]. Prior works have utilized properties of metamaterials such as their deformation from force combined with capacitance to sense changes in response to user interaction [Gong et al. 2021], or to produce non-electronics based interactivity through mechanical interactions [Ion et al. 2017]. Additionally, they allow for interactive machines to be built with a reduced amount of components, in some cases even eliminating the need for assembly [Ion et al. 2016, 2017]. Among this body of work, metamaterial textiles have received considerable attention for their properties including energy absorption and shape-fitting and bending, making them promising for a range of applications [Wang and Hu 2014]. A much studied sub-area of the metamaterial space is textile metamaterials [Wang and Hu 2014], which offer numerous applications in a broad range of areas, ranging from medicine to sports apparel to protective garments [Ali et al. 2018; Kowalczyk and Jopek 2020; Lueling and Beuscher 2019; Moroney et al. 2018; Sun et al. 2021; Wang and Hu 2014].

In this paper, we focus on two types of metamaterials, auxetics and kirigami.

### Kirigami inspired metamaterials.

The Japanese art of kirigami [Callens and Zadpoor 2018], which creates shapes by manipulating a material using cuts and/or folds, can be used to create out-of-plane behavior (i.e., 2.5D shapes). Examples can be seen in Figure 7. Kirigami inspired metamaterials have been used for controlled shaping [Jin et al. 2020; Neville et al. 2016] and to create haptic objects [Chang et al. 2020].

### Auxetic metamaterials.

Auxetic metamaterials are often defined in terms of Poisson’s ratio. The *Poisson’s Ratio v* (eq. (1)), describes how a material deforms perpendicular to applied force [Mihai and Goriely 2017]. *ϵ*_[*x|y*]_ represents strain, defined as the fractional change in length along that axis. The Poisson’s Ratio is typically between 0.5 and −1 for linear elastic materials [Saxena et al. 2016]. *Auxetic* materials have a Poisson’s Ratio < 0, meaning that when *y* increases (i.e., due to stretching), so does *x*. This is the key property of auxetic materials, that when stretched along one axis, they *increase* in size along the other.


(1) $$v=-\frac{\epsilon_{a}}{\epsilon_{b}}$$

Existing research in these domains includes identifying new metamaterial types with specific predictable properties (e.g., [Mizzi et al. 2018]) and developing layout and simulation tools (e.g., [Ion et al. 2016; Vogiatzis et al. 2017]). Since auxetic materials are typically made up of repeating cells of the same pattern, interesting effects have been created by mixing and matching (e.g., [Ion et al. 2016; Martínez et al. 2019]), allowing the creation of applications for metamaterials including mechanical function [Ion et al. 2016], texture [Ion et al. 2018], and information storage [Ion et al. 2017]. For example, by creating cells that support things like rotation, compression, and shear on specific axes, a variety of mechanical objects can be created, from door handles to robots [Ion et al. 2016].

#### 2.1 Textile metamaterials

Some materials, such as wool felt and silk, are naturally auxetic [Kelkar et al. 2020; Verma et al. 2020]. In addition, prior works have created auxetic fibers, foams, and laminates [Wang and Hu 2014] or enhanced the auxetic properties of felt [Verma et al. 2020]. Additionally, modifying textiles through methods such as laser-cutting holes in them can convey auxetic properties (e.g., [Dobnik Dubrovski et al. 2019]). Felts are particularly well-suited to this type of modification because they do not unravel when cut, unlike woven and knitted fabrics. 3D printing on fabric has also been used to create auxetic structures [Shajoo et al. 2021]. Weaving can imbue auxetic properties into woven fabrics (e.g., [Cao et al. 2019; Kamrul et al. 2020; Zulifqar et al. 2018]), as can machine knitting [Ma et al. 2017]. For example, auxetic and self-folding knitted fabrics are both relatively easy to make using the shaping effects created when switching between purl and knit stitches [Knittel et al. 2015; Liu et al. 2010]. Other options include using multiple beds with different interlocked patterns that create an uneven rotational shape [Ma et al. 2016]; using multiple types of fibers [Kamrul et al. 2020; Steffens et al. 2016; Zulifqar et al. 2018]; or using machine-knitting lace techniques to include holes in a pattern [Hu et al. 2011; Ugbolue et al. 2011].

Non-metamaterial shaping effects have also been used in research [Mecnika et al. 2015], such as the creation of 3D structures achieved through gravity by hanging lace structures upside down and then stiffening them [Lehrecke et al. 2021]. Ceron *et al.* embroidered a spiral pattern on a water-soluble stabilizer, which they then encased in silicon to create self-sensing soft inflatable actuators [Ceron et al. 2018]. De Rocha *et al.* used embroidery to create silicone actuators that can be integrated into textiles [Goveia da Rocha et al. 2021]. They combined this with 3D printing on the fabric to create a mold for the silicone bubble which they then inflated by blowing air through a plastic tube that was attached with couching, a form of single-thread stitching. Jo *et al.* developed a variety of embroidered lace items meant to be worn on the body (when attached with hairspray, they last hours) [Jo and Kao 2021].

Despite this rich body of work, there are few examples of embroidery being used to alter the mechanical properties of a material. DIGISEW used anisotropic stitch patterns to control stretch [Sati et al. 2021]. By applying threads using a graded embroidery process that is thinner at the creases, Alharbi *et al.* were able to create a proof-of-concept antenna that could be tuned through folding [Alharbi et al. 2018]. Stoychev *et al.* developed a computational method for predicting and designing the impact of stitch patterns on desired folding behavior of fabric sheets [Stoychev et al. 2017]. Nabil *et al.* developed a method to sew with muscle wire, and demonstrated a variety of shapes including bends, swirls and twists [Nabil et al. 2019]. This allowed them to create an object that could do things such as unroll, or crumple. However, none of these works used metamaterials to alter mechanical properties of embroidered fabrics.

#### 2.2 Working with Lace

To our knowledge, prior work has not investigated the potential for embroidered freestanding lace to create structured mechanical metamaterials. There has been material experimentation with lace to create interactive, light-emitting prototypes with aesthetic properties [Taylor and Robertson 2014]. Such research surfaces a benefit of working with lace- the ability to combine art and functionality and produce subjectively beautiful visual effects [Taylor and Robertson 2014]. Additionally, studies have shown how embroidered freestanding lace can provide a lightweight but durable material flexible enough for on-skin use, even when integrating interactive features [Jo and Kao 2021]. Machine embroidered freestanding lace is created by embroidering a pattern on a water-soluble stabilizer. After washing away the stabilizer, the lace is very strong and slightly stiff, and as a result easily shaped. In the craft community, such lace is often used to create 3D objects such as holiday ornaments. We found one example of a hand-embroidered metamaterial: Granberry *et al.* demonstrated the auxetic properties of handmade needle-lace, which is constructed by wrapping yarn around pins embedded in foam [Granberry et al. 2019]. This helps to demonstrate the potential for machine embroidered freestanding lace to create metamaterials but leaves many open questions about how to do this and what effects are possible to create.

## 3 AUXETICS

There is a broad design space of potential meta materials that can be explored, as summarized in numerous survey papers (e.g., [Askari et al. 2020; Bertoldi et al. 2017; Kelkar et al. 2020; Ma et al. 2017; Saxena et al. 2016; Wang and Hu 2014]). In this section, we report on our experiments with a range of common 2D metamaterial structures and their mechanical properties. Additionally, we explore early-stage design prototypes of functional objects composed of auxetic lace.

### 3.1 Auxetic Lace Design Space Exploration

Auxetic lace is constructed by repeating a cellular pattern that conveys auxetic properties. Although the resulting lace is somewhat stiff due to small residual amounts of the dissolvable substrate lace is printed on during manufacturing, we found empirically that compressing it tended to cause out of plane effects. Thus we focused our exploration on auxetic options that have a negative Poisson’s Ratio when *stretched*. Mechanical auxetics of this type can be divided into three primary groups; examples are shown in Figure 1. Here we summarize Kolken and Zadpoor’s definitions to highlight the range of cell structures that may be amenable to machine embroidery [Kolken and Zadpoor 2017]. Experimenting with this range is important for two reasons. First, it is possible that only some of these will work when machine embroidered. Second, different auxetic structures have measurable performance differences in terms of their Poisson’s Ratio and other factors such as how plastic, or elastic they are, which may impact the types of applications they are well suited for.

#### Re-entrant structures.

**Figure 2:**
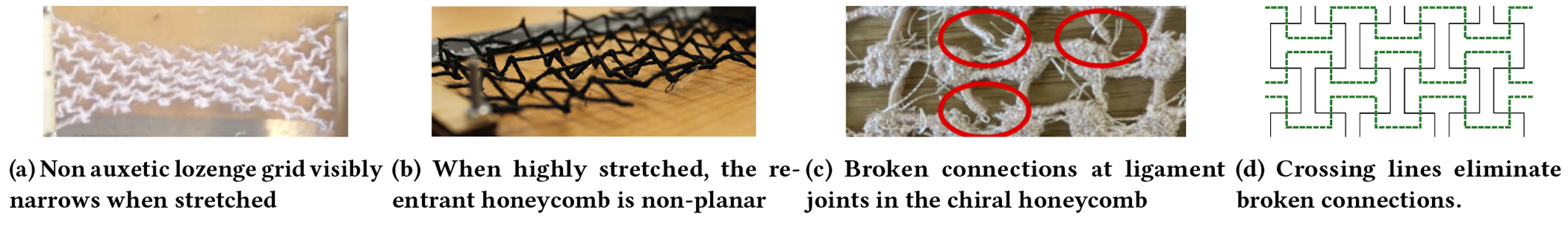
We encountered several different types of errors during our prints.

have an inward angle that can open up. There are several categories including the square grid structure shown in Figure 1a; the lozenge grid shown in Figure 2a; the re-entrant honeycomb shown in Figure 1c and Figure 1c; and the re-entrant triangles shown in Figure 1d. The angle, the length of ligaments, and the thickness of ligaments, all impact the behavior of these structures.

#### Rotating (semi-) rigid structures.

are made up of squares or other rigid shapes connected at the corner by hinges. They often achieve a Poisson’s Ratio of −1 and a range of Young’s modulus values. These can also be constructed by creating perforations in a sheet of material, leveraging flexibility instead of hinges to create motion. An example of rotating squares is shown in Figure 1e.

#### Chiral honeycomb structures.

involve a series of repeating ligaments attached around a central node. The node is expected to rotate when the material is stretched, causing the ligaments to pull on it. An example is shown in Figure 1b. Variations on this approach involve varying which side of the nodes ligaments attach to, while maintaining symmetry. This can modify which dimensions of the material are auxetic. A higher number of ligaments per node increases stiffness, while adding nodes decreases in-plane stiffness (compared to analogous re-entrant structures).

We experimented with embroidered lace versions of each of these types of auxetic structures. Forty-weight Polyester thread (120d/2) was used for embroidery, which was done on a Janome Skyline S9 and a Brother SE1900. Embroidery was done on *Jenny Haskins private selection Dissolve Magic Sticky Fibrous Water Soluble Stabilizer* without adhesive. When a repair was needed mid-print, the same stabilizer with adhesive was added. Stitching was set using the Embrilliance software as described earlier. Post processing consisted of agitating the design in a bowl of water repeatedly for five minutes, and then leaving it to dry and stiffen overnight. Any residual thread caught in the stitches was cut to avoid accidental ties that might impede the design’s flexibility. Depending on the embroidery path, this can be a common issue, and while contributing to a frayed or fuzzy appearance (as seen in Figure 1b), but did not have a structural impact on the finished designs.

Numerous iterations were done to empirically determine which types of metamaterial structures were best suited to manufacturing with an embroidery machine. For example, we found that too acute of an angle resulted in ligaments overlapping and being seamed together in the embroidery process. This could usually be addressed by adjusting a design parameter, such as the angle used in the creation of the re-entrant triangles. Another problem that occurred in our early prints was a pattern that did not hold together after washing. This was most commonly caused when a ligament’s endpoints were not caught securely in the embroidery it was meant to attach to. Three examples of this failure are visible in Figure 2c, marked by red ovals. In most cases, because of the repeating nature of the patterns, it was possible to address this by finding longer overlapping paths through the pattern. For example, our successful square grid pattern (Figure 1a) was constructed of a grid of square waves (Figure 2d). Each dotted horizontal green line, and each solid vertical black line, is a single connected line of stitches. This means that there are no ligament endings that can disconnect at the crossings. Similarly, the re-entrant triangles shown in Figure 1d can be constructed from horizontal lines of connected Vs, with no need for any break in the thread in the horizontal direction within a row. Vertically, the tip of one V must completely overlap the tip of the V in the line below it to ensure the connection is secure. In contrast, we were not able to find a robust way to print the chiral honeycomb. Finally, our consumer-grade embroidery machines were prone to errors such as the top thread being caught and pulled down into the bobbin area. Often this could be traced to a developing problem such as a slightly bent needle, or a tiny nick in the bobbin case. A combination of machine maintenance, and careful work observing prints and halting when any change in sound occurred was used for debugging. Occasionally a hole would develop in our stabilizer material. This was easily fixed by placing additional (sticky) water-soluble material over the damaged area, but sometimes resulted in additional repetitions of embroidery.

We iterated on producing the machine-embroidered metamaterials in this experiment over the span of several years. The demos are still strong and functional. Further, we were able to consistently reproduce both our experimental swatches of auxetics as well as create new versions of the design prototypes throughout these replications. Properties and characteristics of our designs remained consistent when produced on different embroidery machines as previously described and with various brands of forty-weight thread.

### 3.2 Auxetic Lace Empirical Experiments

According to a variety of theoretical (and a few empirical) analyses of in-plane behavior (e.g., [Kolken and Zadpoor 2017]), re-entrant structures consistently have the lowest (most negative) Poisson’s Ratio, while chiral and rotating semi-rigid structures have a Poisson’s Ratio closer to 0. The anti-tetra chiral structure shown in Figure 1b has the best theoretical potential for a low Poisson’s Ratio. Since the measurements may differ based on construction and material, we conducted empirical tests to confirm how our lace structures performed. Our final test set included the square grid pattern shown in Figure 1a; the re-entrant honeycomb shown in Figure 1c; the re-entrant triangles shown in Figure 1d and the rotating semi-rigid squares shown in Figure 1e. We selected designs that did not fray or fall apart when stretched, and that on visual inspection did not obviously have a Poisson’s ratio above 0. This eliminated the chiral honeycomb, which was not robust (Figure 2c) and the lozenge grid pattern shown in Figure 2a, which was not auxetic.

#### 3.2.1 Method.

In all cases, we modeled the lace in SVG format, and then used an off-the-shelf software, Embrilliance with Stitch-Artist, to convert that to stitching instructions. We used the default Embrilliance setting for lace infill to create solid regions (such as the fill for the rotating semi-rigid squares in Figure 1e), and printed borders and lines using: 1.8mm wide satin stitch with forty-volume thread or 1.4mm wide with sixty-volume thread. Unless otherwise specified, borders and lines were printed using the standard Embrilliance settings for freestanding lace infill, which involves multiple layers of inner stitches, with the final layer of satin stitch perpendicular to the direction of the line. The pattern is then printed on a water soluble stabilizer. These settings were chosen to ensure that the stitches do not unravel when the stabilizer is washed away. Each sample was prepared by securing its ends between two pieces of wood with an attached hook. One end was attached to a fixed point, and the other to a digital electronic scale. This setup was placed under a camera on a tripod pointing down at fixed height. The entire loading setup was videotaped during all experiments. The length and height of the entire sample was measured digitally using ImageJ^1^ at rest, and at each level of applied force. Poisson’s ratio was calculated with loads under the level that caused more than 1% plastic deformation. This maximum was determined empirically: An experimenter pulled on the scale, loading it slowly to the value below the plastic deformation, taking measurements in both the stretched and unstretched conditions at each loading level. To determine the percentage of plastic deformation of each material under repeated loading, the experimenter pulled on the tension scale in increments of 0.25lbs/1.1N from 0lb/0N to 1lb/4.4N or 2lbs/8.9N (depending on the material), always releasing the sample between loadings. The overall length that the sample returned to after each repeated loading condition was then measured.

#### 3.2.2 Results and Discussion.

**Figure 3:**
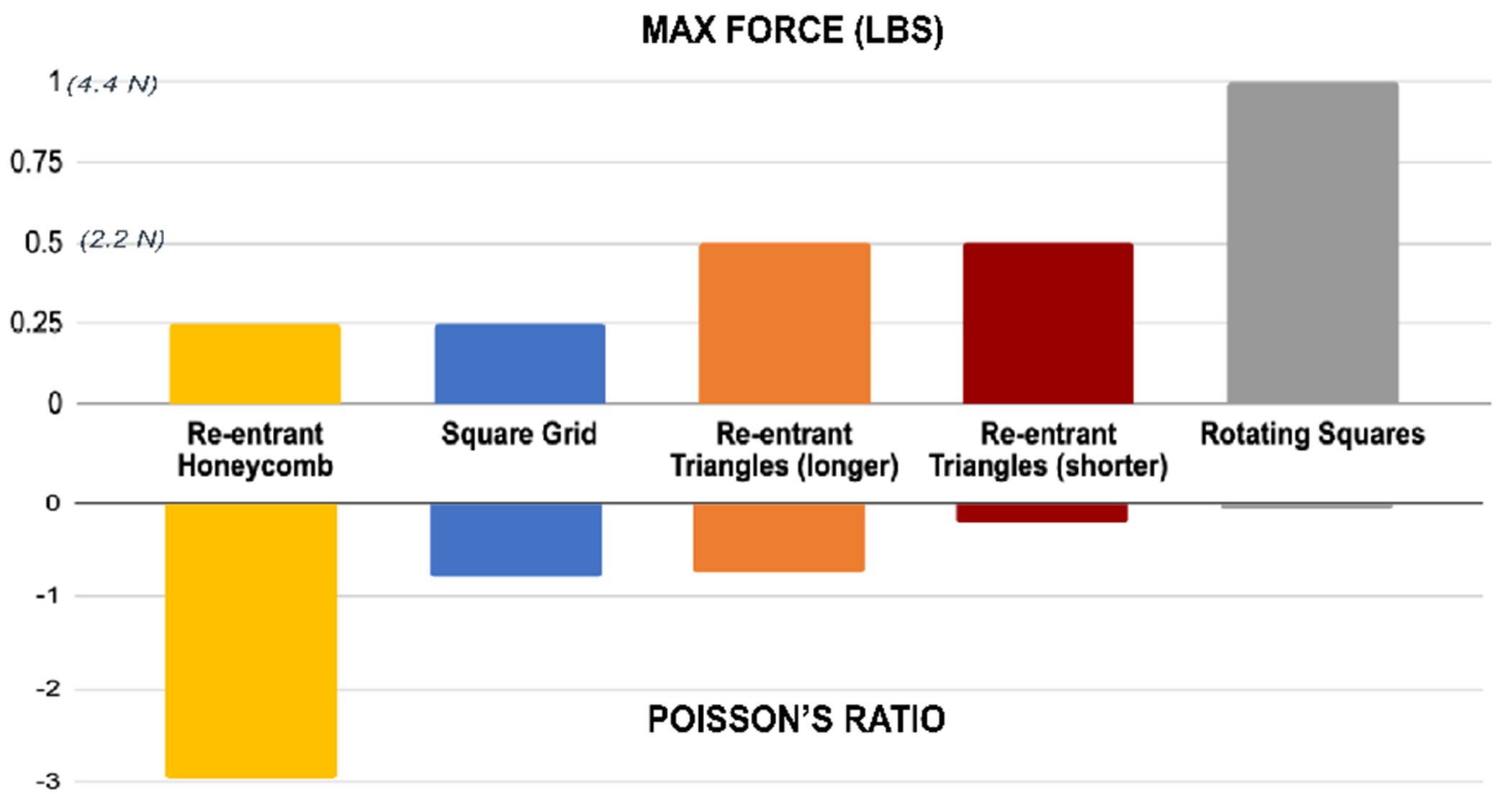
Experimental results. Top bar shows the maximum force before plastic deformation. Bottom bar shows Poisson’s Ratio at that force.

Our results verified the potential for manufacturing auxetic metamaterials using machine embroidered lace. All of the auxetic structures demonstrated strength throughout our experiments, not showing signs of unraveling or other deterioration despite repeated experiments. The re-entrant honeycomb, square grid, both modifications of re-entrant triangles, and rotating squares were all auxetic, however they differed significantly with respect to the maximum force that could be applied without plastic deformation. As shown in Figure 3, the re-entrant honeycomb and square grid both were measured at 0.25 lbs/1.1N, with mean values of Poisson’s ratio of −2.97 and −0.79. The first modification of re-entrant triangles with shorter sides could handle 0.5lbs/2.2N of force with a Poisson’s ratio of −0.21. Increasing the side lengths of re-entrant triangles resulted in a more auxetic sample, with an average Poisson’s ratio at −0.74 for the same force applied. Rotating squares were the least auxetic with a Poisson’s ratio of −0.05 at 1lb/4.4N of force.

The materials also varied significantly in how much they deformed under varying loading conditions. For example, the rotating squares appeared to be the stiffest of all, only starting to exhibit large plastic deformation (over 1% of the original length) after loads of 2lb/8.9N. Re-entrant honeycomb, on the other end of the spectrum, displayed large plastic deformation (over 6% of the original length) with loads of just 0.5lb/2.2N. Further, this pattern exhibited large out of plane deformation when higher loads than 0.5lb/2.2N were applied, as shown in Figure 2b. Changing the length of the re-entrant triangles also appears to change their plastic deformation behavior. While the sample with the longer sides resisted plastic deformation better than its counterpart until about 1lb/4.4N, it yielded significantly higher plastic deformations for loads of over 1lb/4.4N.

We attribute the relatively low Poisson Ratio of the rotating semi-rigid squares to the lack of flexibility at their joints. Acute angles (where the structures intersect) introduce overlap in the stitch path, and corners often have more stitches, both of which impede rotation. This means that the full range of positioning is not available with this semi-rigid structure. We were surprised by how, despite having similar, swastika-shaped components, the lozenge grid and the square grid proved to have different properties. The square grid was consistently auxetic, while the lozenge grid was not. We believe that factors influencing this may include the size of the cells- with the square grid having larger cells than the lozenge grid- and the extent to which the design allowed, or impeded, rotation at the joints. A limitation of our experiment is the variations in the density of each material. Since the minimum size of a stitch is fixed, changes in the size of a pattern could affect results. Future work should repeat experiments with a larger rectangle and fixed grid size. Additionally, this is an early exploration so it is likely that additional design iterations could yield increases of auxetic properties (*e.g.* reducing the density of the cross stitch in the recumbent squares pattern), however, the trade-offs presented by these changes could impact durability and utility and would need to be considered depending on function of the material. Future work should expand on this experiment to test the Poisson Ratio and plasticity of materials in a carefully stratified fashion to better understand their performance characteristics.

### 3.3 Auxetic Forms Exploration

Based on the properties of some of the tested auxetic metamaterials–strong, able to handle deformation without shrinking– we created early-stage prototypes of auxetic forms. We chose re-entrant triangles because they performed well with regard their auxetic properties and did not deform as easily as other options. We used a “research through design process” [Stappers and Giaccardi 2017] for the design space exploration. We created functioning early-stage prototypes of objects constructed out of auxetic lace. Some of the chosen prototypes were re-designs of existing, common objects that offered improved accessibility. Others simply replicate existing functionality present in materials such as spandex or neoprene but offer improvements unique to the medium of embroidered, freestanding lace such as increased breathability, comfort, aesthetics, and ability to integrate with other materials. The chosen auxetic prototypes focus on physical form rather than digital functionality, which will be explored later in the kirigami section of the paper. While we ultimately envision applications that might mix form and function, these demonstrations have value even as static objects.

To support the creation of auxetic forms, we built a web interface with Flask and HTML/JavaScript with a backend Python library extension of svgpathtools that provides support for both re-entrant triangles and a lozenge grid. The library supports standard operations like rescaling and translating shapes, allowing different filled regions to be placed side by side to construct a larger object.

#### Moldable fabric.

**Figure 5:**
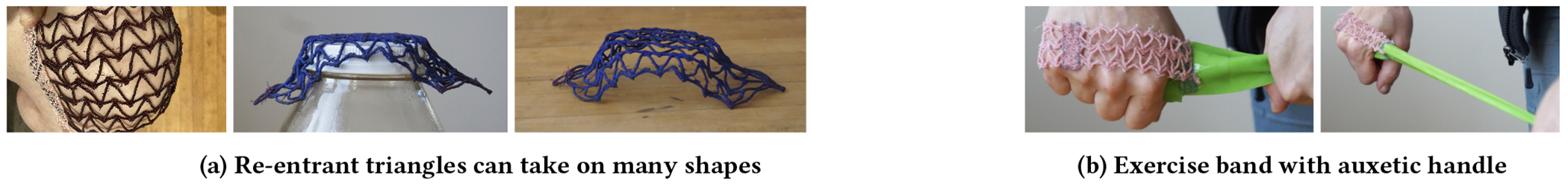
Auxetic forms

We used a unique property of auxetic metamaterials, that they can bend in multiple directions at once, to create a rectangle that will mold to various shapes. We embroidered a rectangle of re-entrant triangles and then coated it in silicone. Figure 5a shows the fit to a knee, and a jar lid. Note how it holds the shape it was molded to even after being removed. This has interesting potential for a post processing step such as transferring the shape to another medium, or hardening it for longer term use. In this case, post-processing the embroidery with silicone enhanced the ability of the material to grip, as well as its ability to hold a shape over time. For uses such as body-forming, the ability to mold with silicone could potentially increase comfort when compared to regular thread for certain uses, such as when utilized for protective knee wear.

#### Exercise Bands.

Exercise bands are inexpensive, highly effective tools for physical therapy [Simoneau et al. 2001]. They are rated according to the amount of physical force needed for 100% elastic deformation of the material [Simoneau et al. 2001]. However, they are flat, slippery, and non-auxetic, causing them to become hard and thin when stretched- and prone to sliding when coming into contact with sweat on skin. Embroidered auxetic material could easily allow for integrated handles that could increase comfort, or even pockets that could fit over a limb that does not have grasping capability, while still allowing for some stretch. Figure 5b shows an exercise band we added a handle to. In the rightmost image, the non-auxetic part of the exercise band is visibly compressed, making it uncomfortable and difficult to hold and requiring a strong grip. In contrast, the handle requires no gripping strength, remains flat due to the fact that its Poisson’s Ratio is negative, and is thus comfortable.

#### Fidget Toys.

**Figure 6:**
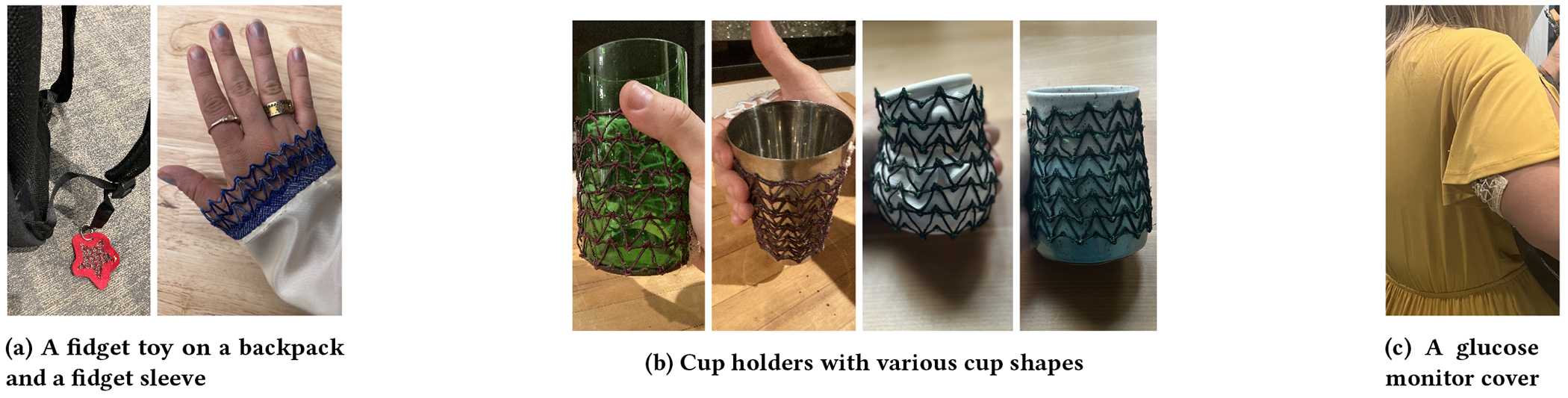
Additional auxetic forms

Fidget toys were popularized in the 2000’s as a helpful tool for neurodiverse people. Typically, they are hand-held items that can spin or move in diverse ways, allowing for stimming. However, as stand alone items, they are both noisy, obvious (potentially identifying a person as neurodivergent), and easy to lose. A responsive embroidered auxetic item creates a unique sensory experience to other fidgets– the ability to deform in ways different from conventional materials can create a stimulating experience. Additionally, the ability to withstand deformation over time without changing shape, as demonstrated by our experiments, makes auxetic fidgets a durable option. Anecdotally, we created an auxetic embroidered fidget that was in use for over a year and preferred in many circumstances to other, noise and more visible alternatives. The embroidered medium allows for these fidgets to be directly integrated into clothing, reducing the risk of losing or forgetting a fidget, which can be a common problem in some neurodivergent conditions [Stormont-Spurgin 1997]. Embroidered fidgets can be more subtle than existing fidgets and be designed in a way to match a person’s wardrobe and style. In addition, the ability to create arbitrary shapes for holding, and to add pockets, could help to match someone’s dexterity, making it more accessible to multiply disabled people. Figure 6a shows an example of an auxetic star. This also demonstrates the ease with which auxetics can be integrated into arbitrarily shaped regions. Figure 6a also shows a fidget integrated into the sleeve of a shirt. This provides a fidget surface hidden in the context of a decorative garment element.

#### Cup Holders.

We made a cup sleeve with a handle, out of auxetic material, so it could fit to a variety of cups. The ability of auxetics to deform in multiple directions allows for a well-fitting grip on the cup, even if a cup size or form is unusual. This allows for accommodating a variety of styles with a single holder such as mugs, wine glasses, pint glasses, and more. Existing cup sleeves with handles commercially available tend to be made from non-auxetic materials and only accommodate a specific cup shape type [Amazon 2024a,b,c], which requires an individual to bring all of these holders along to a dining place where they may choose to have multiple types of beverages in different cups. An auxetic alternative allows for more flexibility with a single cup sleeve. We empirically determined tube size to accommodate a variety of cup shapes (cylindrical and cone-shaped) and sizes, and added a handle. Cups without handles can be difficult to hold for some people with mobility disabilities. Figure 6a shows the form-fitting flexibility of auxetic cup holders on cylindrical, conic, and unevenly shaped cups. This is an easy, portable and lightweight solution, allowing someone to add a handle to various cups they may come across.

#### Glucose Monitor Cover.

We made a cover for a continuous glucose monitor (CGM). CGMs are commonly worn on the arm and come in various shapes and oftentimes have attachments for functionality such as connectivity that result in irregular, bulky shapes. Existing spandex covers exist but are not designed with auxetic properties and do not accommodate irregularly-shaped attachments by default and some of these options cause skin issues such as irritation and excessive sweating due to lack of breathability. Our auxetic, embroidered prototype conforms to both (1) the shape of a Dexcom G6 with an attached sensor and (2) to a curved arm, without excess pressure, cutting in, or shrinking. We used double-sided body tape to attach it for the preliminary prototype. The prototype shown in Figure 6c was comfortable, breathable, and durable enough to wear throughout the day and night, and presented an alternative to spandex covers that matched better with short-sleeve non-athletic garments such as dresses.

## 4 KIRIGAMI

In this section, we detail our work on using embroidered, freestanding lace to create kirigami patterns and demonstrations. We describe our explorations with twists, cuts, and folds. We then present demonstrations of basic, electrical components designed from conductive threads and prototypes of functional antennas.

### 4.1 Kirigami Lace Design Space Exploration

**Figure 7:**
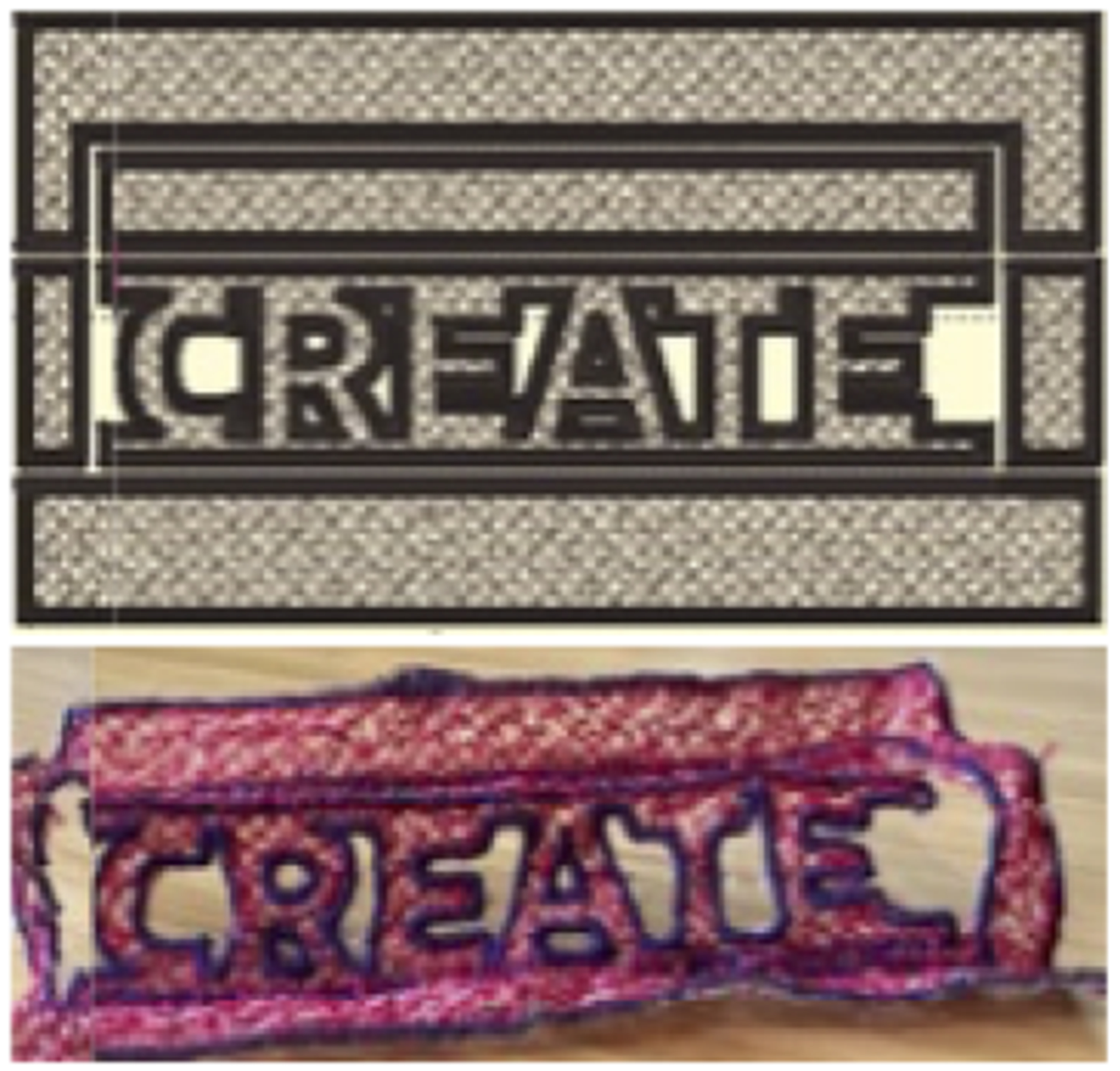
Pop-up letters

We experimented with three types of kirigami design patterns: Twists, cuts and folds. For twists, we replicate [Attard et al. 2017], which is also used in [Chang et al. 2020] and demonstrate two variations, a square and a hexagon. Our approach to cuts and folds draws inspiration from craft embroidery projects such as a kirigami inspired star of Bethlehem [Library 2022]. An example of a pattern using cuts and folds, along with larger regions filled with freestanding lace and outlined with satin stitch set to 1.8mm was used to embroider a pop-out of the word “create” in uppercase letters Figure 7(f).

#### Cuts.

Embroidered satin stitch is typically centered on the SVG pattern line, so a narrow cut, accounting for error, requires about a 3mm gap to avoid overlap. We used 3mm rectangular hole to model cuts in our SVG files, and outlined them with 1.8mm satin infill for printing.

#### Folds.

We created fold lines by adding the same 3mm wide hole at the fold, but added a second identical rectangle to fill with connecting stitches. This second rectangle is filled with a zigzag column of 3mm width, creating a series of connected cross bars that would easily bend but still hold the two solids together.

#### Twists.

**Figure 8:**
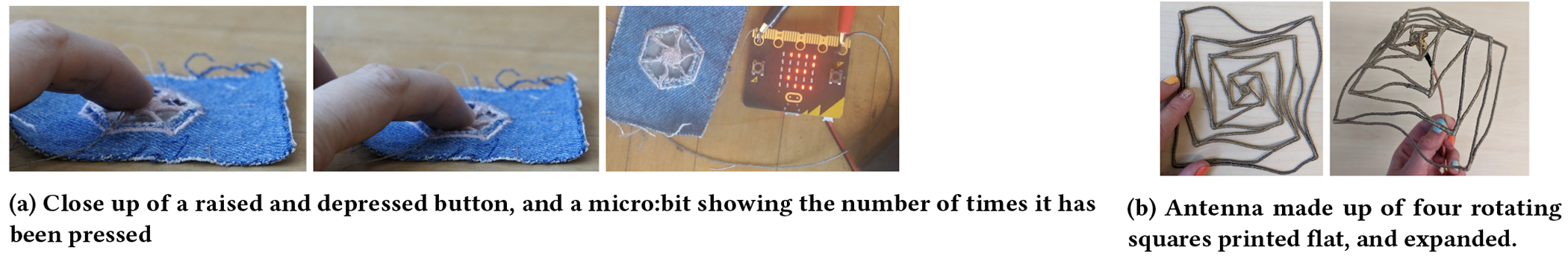
Basic Components

Twists are a property of the SVG model. Two solids (such as a square or hexagon) are nested concentrically. The corners of the inner and outer solid are connected by ligaments in a rotating pattern so that corner 1 (outside shape) is connected to corner 2 (inside shape), and so on. When the inner shape is untwisted after printing, it rises up to accommodate the length of the connecting legs. As can be seen in Figure 8b this effect can be nested, producing fairly large changes in height from a flat material.

### 4.2 Kirigami Demos: Basic Electronic Components

We first show that freestanding lace metamaterials can be used to create basic electronic components. A variety of common electronic capabilities have been replicated in the past, using sewn conductive thread. Some examples include wireless power [de Medeiros et al. 2021; Sun et al. 2020]; contactless EMG sensing [Linz et al. 2007]; pressure sensing [Aigner et al. 2020; Post et al. 2000; Wu et al. 2019]; touch sensing (e.g., [Jo and Kao 2021; Roh 2017]); proximity sensing [Gilliland et al. 2010]; tilt sensing [Zeagler et al. 2012]; embroidered speakers made with both sequins [Zeagler et al. 2012] and conductive thread [Preindl et al. 2020]; pressure sensing [Parzer et al. 2018]; NFC or RFID tags (or more specifically, their antennae [Jiang et al. 2019; Jo and Kao 2021; Ukkonen et al. 2012; Wang et al. 2021]); photovoltaic [Yu et al. 2021] and RF [Vital et al. 2020] power harvesting; electrodes [Trindade et al. 2014]; recognizing conductive objects using inductive coils [Gong et al. 2019]; transistors [Hamedi et al. 2009]; and embroidered printed circuit boards [Buechley and Eisenberg 2009]. A separate thread of research has explored how to replicate interactive components, including a button [Goudswaard et al. 2020]; rocker switch [Gilliland et al. 2010]; menu [Gilliland et al. 2010]; slider (using a zipper [Gilliland et al. 2010]); jog wheel [Zeagler et al. 2012]; keyboard [Orth et al. 1998; Post et al. 2000]; touchless gesture recognition [Wu et al. 2020]; and stuffed VR interactors [Tyagi 2018]. We add to this body of work by demonstrating how embroidery can easily integrate conductive thread into metamaterials to create a button and an antenna.

#### Button.

As mentioned in Section 3, it is possible to embroider a material that, when twisted, rises above the plane of the fabric. Figure 8 shows an application of that approach for creating a button. This demonstrates the potential for embroidery to be integrated into other types of textiles; as well as the compatibility of conductive thread and embroidered metamaterials.

To manufacture the button, we laser-cut a hole in cotton twill where the button would be embroidered. We then placed a water-soluble, sticky stabilizer into the embroidery hoop and laid the laser cut fabric in place. Placement can be verified by pre-printing an outline of the hole on the stabilizer. The kirigami structure was then embroidered in place over the hole, using conductive bobbin thread for the central hexagon. Embroidering with conductive thread required using a metal needle, and reducing the tension on both the bobbin and top thread. Once the stabilizer was dissolved, we sewed conductive fabric to the back of the twill. This allowed us to construct a resistive switch. When the button is depressed and makes contact with the conductive backing, it completes the circuit. Initially, the button returned to its above-plane position with no underlying support, but after repeated testing it eventually stopped springing back. We addressed this by placing a small piece of carbon impregnated conductive polyurethane foam (anti-static foam) between the button and the base layer of fabric, thus increasing the longevity of the switch effect to match the longevity of the foam.

#### Antenna.

In the past few years there has been increasing experimentation with e-textile and wearable antennas using a variety of techniques and geometries (e.g., [Alharbi et al. 2018; Doan et al. 2016; Lewis 2020; Psarra and Briot 2019]). One approach to creating wearable, RFID antennas even utilized embroidering copper wire onto textile substrates such as felt and wool to create designs that could have aesthetic value- such as embedding the RFID into an embroidered logo [Doan et al. 2016] onto clothing. However, to our knowledge there have not been experiments with freestanding lace antennas prior to this work. We created a simple kirigami textile antenna using conductive thread. Specifically, the embroidered shape contains four nested squares connected in a twisted pattern, which allows for the antenna to expand vertically. The center of the twists becomes the point of connection where we added an SMA coaxial connector, which allows for the antenna to be connected to a testing device. The antenna was manufactured using Madeira HC-40 conductive thread both on the top and bottom bobbin of a Brother Persona digital embroidery machine, on Vilene water-soluble stabilizer from Allstitch.com.

We tested the prototype with an RTL-SDR software-defined radio dongle (a digitally tuned radio device) and the CubicSDR software. It demonstrated excellent reception for the FM frequency range (88-108 MHz), and also very good reception for the weather satellites transmitting at 137MHz. Moreover, the antenna was tested with a NanoVNA vector network analyzer using both the standing wave ratio (SWR) and the reflection coefficient (S11 value), which demonstrated multiband qualities, as it was also resonant at about 300MHz, and 1.4-1.8GHz. More details can be found in [Kaputa and Psarra 2023b]. This approach has the potential to be integrated into wearables. Further, if actuated to change shape [Kaspersen et al. 2019], it is possible the antenna may change its resonant frequency, allowing for dynamic tuning. The antenna is extremely lightweight, which could allow for use in settings where weight is a concern, such as outer space applications.

## 5 CONCLUSION AND FUTURE WORK

We have demonstrated the potential for machine embroidered freestanding lace to be useful in creating mechanical metamaterials. Our contributions lay the practical foundation necessary for creating mechanical metamaterials on commercially-available embroidery machines. Our experiments and explorations show that machine embroidery has the potential to create a range of auxetic metamaterials. Our exploration of auxetics also found that auxetic materials that can be expressed using continuous lines that create a grid are especially well suited to machine embroidered freestanding lace. Our demonstrations include a range of basic capabilities and applications. Our experiments with kirigami were generally fruitful, and we believe there is a large space still to be explored in that domain including exploring further interactivity through more complex designs using conductive thread. The recent enhancements of the kirigami antennas confirm there is potential to explore more sophisticated form factors as well [Kaputa and Psarra 2023b]. Electronic applications of metamaterials contribute to the current on-going discussion in the field of e-textiles, and interaction design on the relationship between form and function, as well as material manipulation and aesthetics [Posch 2017].

The potential uses of these materials are quite wide-ranging, with many applications in the accessibility space. In addition to those explored in this paper, there is an opportunity to create custom harnesses, such as those used to help hold a person in place in a mobility device; clothing that fits a variety of body shapes, or is more accessible to take on and off (such as an accessible binder or a sleeve that fits over a cast); hammocks; and pockets. One could also explore coating of embroidered structures in a stiffener for the purpose of creating splints and other hard shapes that might be useful in clinical settings. Likewise, future works could examine combining auxetics, aesthetics, and interactivity, redesigning bulky form factors for CGMs such as third-party connected attachments, or adding new functionality such as indicating status or notifying the wearer of an important blood sugar event.

### Limitations and Ethical Considerations.

Additional work is needed to compare the example applications we created to existing solutions. One important consideration in the creation of textiles is e-waste. Some works have begun to explore mitigating waste in the context of e-textiles [Jones 2021; Wu and Devendorf 2020]. A related consideration is durability. Few projects have explored how e-textiles respond to things like washing (an exception is [Zeagler et al. 2013]). However, some of our demos have been utilized for over a year, suggesting durability is not a large concern for machine embroidered lace. Additionally, there is opportunity to further explore whether our results can be replicated with sustainable threads composed of hemp, silk, or cotton in the future– all of which are more sustainable than polyester.

Some of our examples served the purpose of modifying existing objects to make them more accessible. However, we must acknowledge that the tools that we used to create these materials are all off-the-shelf graphic design tools and embroidery tools which are not themselves accessible, outside of our python library– which still cannot result in an end-to-end print without utilizing a tool such as Embrilliance. The lack of accessible design tools for creating freestanding lace metamaterials presents challenges to disabled makers hoping to embroider their own designs or add modifications to standard ones. A good opportunity for future work would be to create tools that provide a more accessible experience for machine embroidery of freestanding lace metamaterials– or overall for manufacturing in general. Enabling accessibility in the design process of freestanding lace metamaterials– given the available, ubiquitous nature of embroidery machines– is key for achieving the vision of enabling disabled makers to create their own modifications of existing products as we demonstrated is possible in this paper.

## Figures and Tables

**Figure 4:**
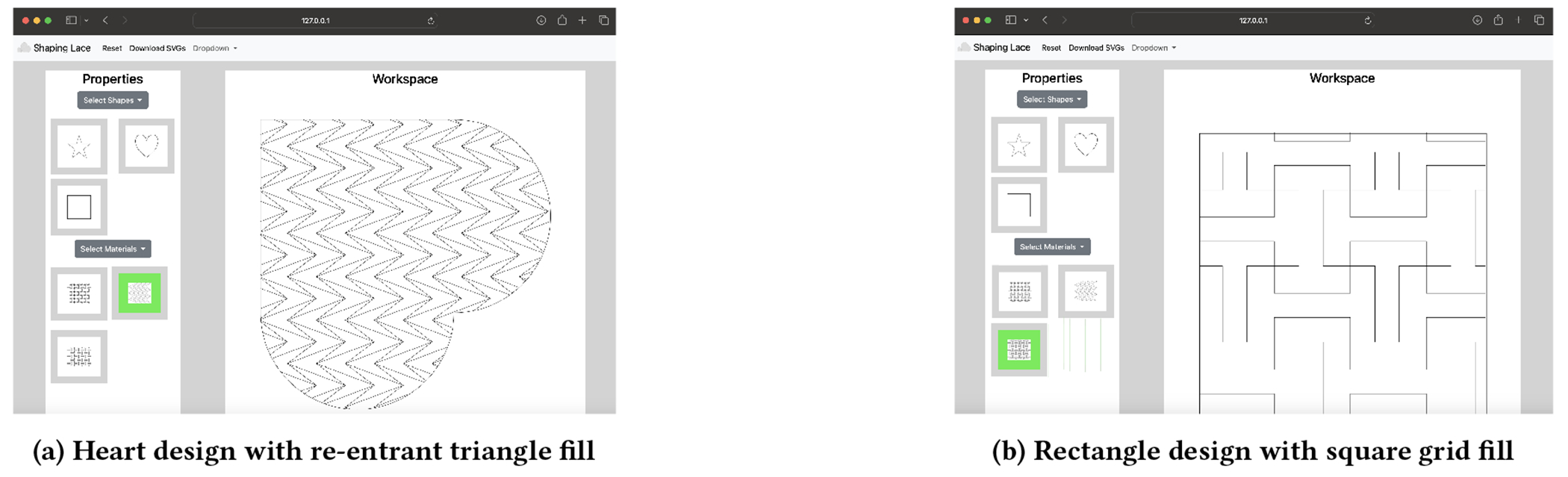
Metamaterial design WebUI

## CCS CONCEPTS

- • **Applied computing → Computer-aided manufacturing**; • **Human-centered computing** → *Accessibility technologies*.

## Footnotes

1
https://imagej.nih.gov/
